# Multi-Level Effects of Low Dose Rate Ionizing Radiation on Southern Toad, *Anaxyrus* [*Bufo*] *terrestris*

**DOI:** 10.1371/journal.pone.0125327

**Published:** 2015-04-30

**Authors:** Karolina Stark, David E. Scott, Olga Tsyusko, Daniel P. Coughlin, Thomas G. Hinton

**Affiliations:** 1 Department of Ecology, Environment, and Plant Sciences, Stockholm University, Stockholm, Sweden; 2 Savannah River Ecology Laboratory, University of Georgia, Aiken, South Carolina, United States of America; 3 Department of Plant and Soil Sciences, University of Kentucky, Lexington, Kentucky, United States of America; 4 Department of Radioecology, Environmental Modeling and Ecotoxicology, Institute of Radiation Protection and Nuclear Safety, Cadarache, France; ENEA, ITALY

## Abstract

Despite their potential vulnerability to contaminants from exposure at multiple life stages, amphibians are one of the least studied groups of vertebrates in ecotoxicology, and research on radiation effects in amphibians is scarce. We used multiple endpoints to assess the radiosensitivity of the southern toad (*Anaxyrus* [*Bufo*] *terrestris*) during its pre-terrestrial stages of development –embryonic, larval, and metamorphic. Toads were exposed, from several hours after oviposition through metamorphosis (up to 77 days later), to four low dose rates of ^137^Cs at 0.13, 2.4, 21, and 222 mGy d^-1^, resulting in total doses up to 15.8 Gy. Radiation treatments did not affect hatching success of embryos, larval survival, or the length of the larval period. The individual family variation in hatching success of embryos was larger than the radiation response. In contrast, newly metamorphosed individuals from the higher dose-rate treatments had higher mass and mass/length body indices, a measure which may relate to higher post-metamorphic survival. The increased mass and index at higher dose rates may indicate that the chronic, low dose rate radiation exposures triggered secondary responses. Additionally, the increases in growth were linked to a decrease in DNA damage (as measured by the Comet Assay) in red blood cells at a dose rate of 21 mGy d^-1^ and a total dose of 1.1 Gy. In conclusion, the complex effects of low dose rates of ionizing radiation may trigger growth and cellular repair mechanisms in amphibian larvae.

## Introduction

From a global biodiversity standpoint amphibians are organisms of concern because many species are threatened and declining world-wide [1–2]. Amphibians are important organisms that can influence the overall energy, nutrient and contaminant dynamics within ecological systems [3–4], and they are key components of many wetland ecosystems, often having the greatest abundance among vertebrates. Over a 1-yr period > 360,000 juvenile amphibians emigrated from a single 10-ha wetland in South Carolina, USA [5]. Because many contaminants can accumulate in wetlands [6–8], most pond-breeding amphibian species may be exposed at different stages over their entire life cycle. Almost all frog and toad species in the US are aquatic breeders [9], which, in the course of their biphasic life cycle, 1) lay shell-less eggs that undergo external embryonic development from a few days to several weeks in shallow standing water, 2) have aquatic larvae (tadpoles) that are exposed to aqueous conditions for several weeks to >1 yr, 3) undergo dramatic morphological reorganization during metamorphosis as they change from a gilled aquatic herbivore to a lunged terrestrial carnivore, and 4) have a scale-less permeable skin for gas and ion exchange that can also directly absorb contaminants [10–11]. These four traits may make them particularly susceptible to environmental contamination (but see [12]). Despite their potential vulnerability to contaminants, historically amphibians and reptiles are the least studied groups of vertebrates in ecotoxicology [13].

Radionuclides and radioactive releases can potentially be harmful to biota in the environment. International research on radiological protection of animals and plants indicates that relevant data for amphibians are particularly sparse, especially from chronic low-dose-rate radiation exposures [14]. Also, the need for further understanding of low-dose impacts on the environment has been recognized by several research groups [15–16]. However, the effects of low dose rates are complex in any living organism and radiation effects data derived from field studies of low-dose radiation can be challenging to interpret and is the subject of debate [17].

The International Atomic Energy Agency, IAEA, found little evidence of population-level impacts to aquatic organisms exposed to radiation at dose rates <10 mGy d^-1^ (1 Gy = 1 Gray; absorption of one joule of energy per kilogram) [18]. Although not originally intended as such, the IAEA value of 10 mGy d^-1^ evolved into an implied regulatory guidance. More recent reviews by the International Commission on Radiological Protection, ICRP, suggest that effects could be considered at dose rates of 1–10 mGy d^-1^ for a reference frog that is one of 12 reference organisms in the ICRP environmental radiation protection system [19–20]. In general, few data exist on translating sub-lethal radiation effects from individuals to population-level responses [14].

To start to fill the current knowledge gaps concerning low dose radiation effects on amphibians, we performed an experiment with the hypothesis that the IAEA dose-rate guidance of 10 mGy d^-1^ for aquatic populations is sufficient to protect amphibians. To test this, we exposed eggs and larvae of an amphibian species, the southern toad (*Anaxyrus* [*Bufo*] *terrestris*), to low dose rate ionizing radiation from ^137^Cs sources. To increase the realism of environmental conditions during the exposures, which lasted up to 77 days, we used an outdoor exposure facility as described in detail in a previous study [21].

We were particularly interested in assessing amphibian radiosensitivity during embryo, larval, and metamorphic periods when rapid cell division occurs. Dividing cells are more sensitive to ionizing radiation than non-dividing cells, and exposure to ionizing radiation during cell division can lead to abnormal mitosis, growth, or metabolism [22]. In the course of the irradiation treatments, we measured individual-level responses that could have implications on population dynamics, such as hatching success of eggs/embryos, larval survival, and age (= larval period) and body size at metamorphosis [23–24]. The duration of the larval period and metamorphic timing largely influences the recruitment of amphibian juveniles [25–26], and body size at metamorphosis can influence reproductive success later in life [23]. In addition, at the cellular level, we measured DNA damage after low dose rate exposure to examine whether the level of DNA damage and cellular repair mechanisms coincided with radiation effects on other response variables such as growth during development. To our knowledge, a link between DNA damage and population effects has not been determined for non-human biota [27].

## Materials and Methods

### Study Species

*Anaxyrus* [*Bufo*] *terrestris* is a common toad species in the southeastern United States. On average, female *A*. *terrestris* lay 2,500–4000 eggs attached in coils to vegetation in shallow water [28] (Table 1). Depending on temperature, the eggs hatch within 2–4 days and the larvae develop for 30–63 days before metamorphosis [9]. In prior studies on *A*. *terrestris*, average snout-to-vent length (SVL) at metamorphosis was 7.5 mm [29] and average wet mass ranged from 70–130 mg [30].

**Table 1:** Life history characteristics of Southern toad.

Species	Common name	Egg development (days)	Larval period (d)	Metamorph SVL^1^ (mm)	#Eggs per female	Size at maturity (cm)
*Anaxyrus terrestris*	Southern toad	2–4	30–63	7.5	2,500–4,000	4–9

Typical key life history data [9] for the amphibian species studied.

^1^ snout-to-vent length.

### Experimental setup

In the exposure experiments we used an outdoor low dose rate irradiation facility (LoDIF) at the University of Georgia’s Savannah River Ecology Laboratory (SREL) [31]. The outdoor facility consists of 40 fiberglass, open-air mesocosms (each mesocosm is 2.4 m in diameter, 41 cm maximum water depth) that are used to rear aquatic organisms. Water from a nearby lake is pumped to all mesocosms in a flow-through system. The facility is divided into eight replicated blocks, with each block containing two mesocosms without a radiation source and three with ^137^Cs sources of three activities (0.74, 7.4 and 74.0 GBq) mounted above them, producing nominal dose rates of 1, 10, and 100 mGy d^-1^. Thus, the LoDIF is ideally suited to test the IAEA guideline of 10 mGy d^-1^. To expose tadpoles in a smaller area and to a more homogenous radiation field, we confined them within each mesocosm to 19-L experimental buckets placed directly below the ^137^Cs source; embryos were further restricted to a uniform water depth in smaller (3 L) buckets within the larger buckets. Thermoluminescent dosimeters were placed in the buckets to measure dose rates during each stage (embryo and larval exposures) of the experiments. Control buckets for tadpoles, without a ^137^Cs source, received an average dose rate of 0.13 mGy d^-1^ due to radiation scatter from nearby irradiated mesocosms within each block. Even though 0.13 mGy d^-1^ is above background we refer to this treatment as control dose rate because irradiated tadpoles received dose rates 1–3 orders of magnitude higher, with average dose rates of 2.4, 21, and 222 mGy d^-1^. In the smaller buckets used for embryo exposures, dose rates averaged 0.17, 3.6, 32, and 343 mGy d^-1^. Additionally, the dose rate received by the controls (5–7 μGy h^-1^), was less than the 10 μGy h^-1^ thought to be a screening benchmark value below which ecosystems are protected from ionizing radiation [32].

In addition to periphyton growing in the experimental buckets, tadpoles were fed ground TetraMin fish flakes (48% protein) two to three times per week. All radiation sources were turned off during feeding, collection of metamorphosed juveniles, and any other subsampling or system maintenance. In total, the sources were turned off less than 3% of the exposure time. This time when sources were turned off were not taken in to account when calculating total doses from exposure. Tadpoles were exposed until they reached metamorphosis, defined here as the point after forelimb emergence when the tail is resorbed and the physical appearance is that of an adult (developmental stages 45–46; [33]. Water temperatures in the experimental tanks were monitored, and the tank means ranged from 23°C in May to 29.5°C in July.

### Exposure protocol

Amplectant pairs of *A*. *terrestris* were collected on May 18^th^ (1 pair) and June 1^st^, 2005 (2 pairs) from a reference site, Ellenton Bay (Latitude 33.221631, Longitude -81.747023), on the Savannah River Site (SRS). Adults collected at the earlier date bred and oviposited naturally (clutch 1); whereas the toads collected at the latter date were injected with human chorionic gonadotropin (100 IU for males and 250 IU for females) to induce mating and oviposition (clutches 2 and 3). We divided eggs in each clutch into subgroups containing ~100 embryos, placed each subgroup in a 3-L hatching bucket, and randomly assigned buckets to the four radiation treatments (0.17, 3.6, 32, and 343 mGy d^-1^) within the LoDIF. For clutch 1 we had four replicates per treatment (i.e., one bucket per tank across four blocks, N = 16); for clutches 2 and 3 we also used four replicates per treatment (i.e., one bucket per clutch per tank across four blocks, N = 16 per clutch). In total, embryos with twelve replicates of each radiation treatment were exposed for three to four days until hatching (N = 48). Total doses to the embryos ranged from 0.5 mGy to 1.4 Gy, depending on the treatment (Table 2). After the completion of the embryo exposures, 30 newly hatched larvae were transferred to 19-L buckets (larval density = 2 individuals/L) and exposed at slightly lower dose rates (0.13, 2.4, 21, and 222 mGy d^-1^) until metamorphosis; embryos from clutch 1 were divided into two buckets with 30 larvae in each resulting in a total of sixteen replicates per radiation treatment (N = 64). We measured the hatching success of embryos, larval survival, larval period, and SVL and body wet mass at metamorphosis. We calculated body index at metamorphosis as mass/SVL, a relative index of body condition. We calculated hatching success as the number of embryos that successfully hatched divided by the initial egg number, which we determined from photographs of eggs in each bucket taken at the start of the treatments.

**Table 2:** Details for the experiment conducted on the amphibian species *Anaxyrus* [*Bufo*] *terrestris*.

Species	Life stage	Dose rate mGy d^-1^	Exposure duration (d)	Dose range (Gy)	Density (animals L^-1^)	Endpoints^a^
*Anaxyrus terrestris*	Egg stage (14–17)	0.13 (0.17)	25–77	0.0033–0.010	2.0 (N = 30)	1, 2, 3, 4, 5
*Anaxyrus terrestris*	Egg stage (14–17)	2.4 (3.6)	20–77	0.048–0.18	2.0 (N = 30)	1, 2, 3, 4, 5
*Anaxyrus terrestris*	Egg stage (14–17)	21 (32)	25–63	0.52–1.3	2.0 (N = 30)	1, 2, 3, 4, 5
*Anaxyrus terrestris*	Egg stage (14–17)	222 (343)	20–71	4.4–15.8	2.0 (N = 30)	1, 2, 3, 4, 5

Life stage describes stage [33] at the start of radiation exposure. Dose rates (mGy d^-1^) in brackets are initial dose rates for eggs/embryos in small buckets. All other dose rates are average rate to tadpoles in larger buckets. Dose (Gy) range is the total radiation dose received during the exposure. The density column refers to animals per liter water, and endpoint numbers are explained below.

^a^ Endpoint explanation: 1) body mass (g), snout-to-vent length (mm), and body index (g/mm) at metamorphosis (stage 45–46); 2) age (days) at metamorphosis; 3) percent (%) survival to metamorphosis; 4) hatching success of embryos (proportion hatched eggs); 5) strand breaks in DNA in red blood cells analyzed with the Comet Assay.

In addition, we examined the level of DNA damage in toads that had been irradiated throughout their entire embryo and larval development periods. Sixteen newly metamorphosed toads were sampled 52 days after start of exposure, and we used the alkaline Comet Assay (described below) to quantify DNA damage in red blood cells.

### Ethics Statement

Collections of animals were made under annual South Carolina scientific collecting permits approved by the South Carolina Department of Natural Resources, and exposure procedures approved by the University of Georgia Animal Care and Use Committee IACUC No. A2005-10169.

### DNA damage

To examine whether low-dose rates of radiation caused DNA damage, we collected blood samples from metamorphosed *A*. *terrestris* and analyzed for single and double DNA strand breaks using the alkaline single-cell gel electrophoresis method (Comet Assay) following the protocol of [34], with the modification of using gelbond film [35]. Blood samples were collected by cardiac puncture from newly metamorphosed toads anaesthetized with MS-222 immediately after radiation exposure, and samples were kept on ice. The red blood cell concentration was calculated by using a hematocytometer, and after serial dilutions with phosphate-buffered saline (PBS), the final cell count used was 300,000 cells/ml. The diluted blood solution (80 μl) was mixed with 80 μl of 1% low melting point (LMP) agarose and transferred on to the gelbond film. After the agarose polymerized, we placed prepared slides in lysing solution (2.5 M NaCl, 100 mM EDTA, 10 mM Trizma base, 1% Triton X-100 and 10% DMSO, pH 10) and left them overnight. After rinsing gently three times with distilled water, we transferred the slides to the DNA unwinding buffer (1 mM Na_2_EDTA, 300 mM NaOH, pH 13) for 15 min, subjected them to electrophoresis at 4°C (25 V for 10 min), and washed them twice with the neutralization buffer (1 x TAE). After two hours of fixation in absolute ethanol and drying, we stained the slides with 80 μl of ethidium bromide (0.04 mg/ml), and analyzed them under an epi-flourescent microscope using Image Analysis software [36]. Parameters measured were percentage (%) of DNA migrated in the comet’s tail and length of the comet tail. A total of 50 cells were scored blindly for each sample.

### Data analysis

Software STATISTICA (StatSoft version 10) was used for all analyses. Because we had multiple (non-independent) tadpoles per bucket, we used, for each parameter, the mean value in each experimental bucket to avoid pseudoreplication [37]. The Shapiro-Wilk W’s and Levene’s tests were used to test normality of data and homogeneity of variances, respectively. When needed, data were log or arcsine square root transformed to better meet normality assumptions. A probability of α ≤ 0.05 (two-tailed) was considered statistically significant. Because there was no block effect (*p* ≥ 0.436; the eight blocks in the facility), data were combined for the different treatments. For embryos in the experiment, two buckets were excluded from clutch 1 because of dragonfly predation, and four buckets were excluded from clutch 3 because eggs showed no development in these buckets, presumably because they were unfertilized. In addition, 20 buckets of tadpoles were excluded from the larval analyses because of predation by dragonflies or unfertilized eggs earlier in the experiments causing density effects resulting in a total of 38 remaining buckets for endpoints measured after metamorphosis (larval period, body size).

We compared hatching success of embryos, larval survival, and larval period between radiation treatment and clutches using factorial ANOVA and Tukey’s HSD and Bonferroni post-hoc test. Differences in body size (mass, SVL, and body index) at metamorphosis among radiation treatments were analyzed with a one-way ANOVA. Because we predicted an ordered response on body size with increasing radiation treatment, the ANOVA tested specifically for a linear trend. Also, we computed the correlation between total dose received during the exposure period and body index. In a supplementary ANOVA, we tested whether the linear trend of radiation treatment varied for the different clutches. To that end, we performed a factorial ANOVA with clutches and the linear effect of radiation treatment on body size. Further, we compared DNA damage among radiation treatments with a one-way ANOVA and Tukey’s HSD post-hoc test. Finally, we computed the correlation between DNA damage and total dose received during the exposure period.

## Results

**Fig 1:**
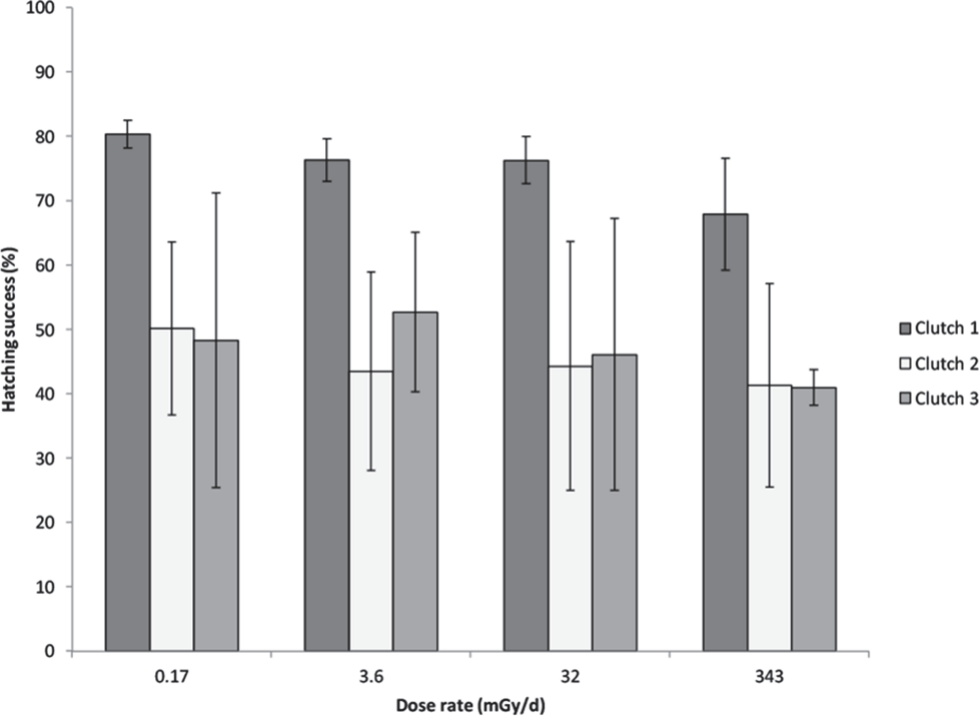
Hatching success of embryos. Hatching success for three clutches of *Anaxyrus* [*Bufo*] *terrestris* embryos exposed for 10 days to low dose ionizing radiation from ^137^Cs. The four different radiation treatments (mGy d^-1^) are indicated on the x-axis. The error bars indicate standard errors of the means.

After exposure to the four radiation dose rates, the proportion of successfully developing embryos of *A*. *terrestris* did not differ among treatments (*F*_3, 30_ = 0.24, *p* = 0.866; Fig 1). In contrast, hatching success was different among the three clutches (*F*_2, 30_ = 4.65, *p* = 0.017; Fig 1) with higher embryo hatching success in clutch 1 than in clutch 2 (Tukey HSD, *p* = 0.023). The interaction between radiation treatment and clutch was not significant (*F*_6, 30_ = 0.022, *p* = 0.999). Recall that clutch 1 was obtained two weeks earlier after natural breeding and oviposition, while clutch 2 and 3 were obtained after induction of oviposition and fertilization by injection of human chorionic gonadotropin.

**Fig 2:**
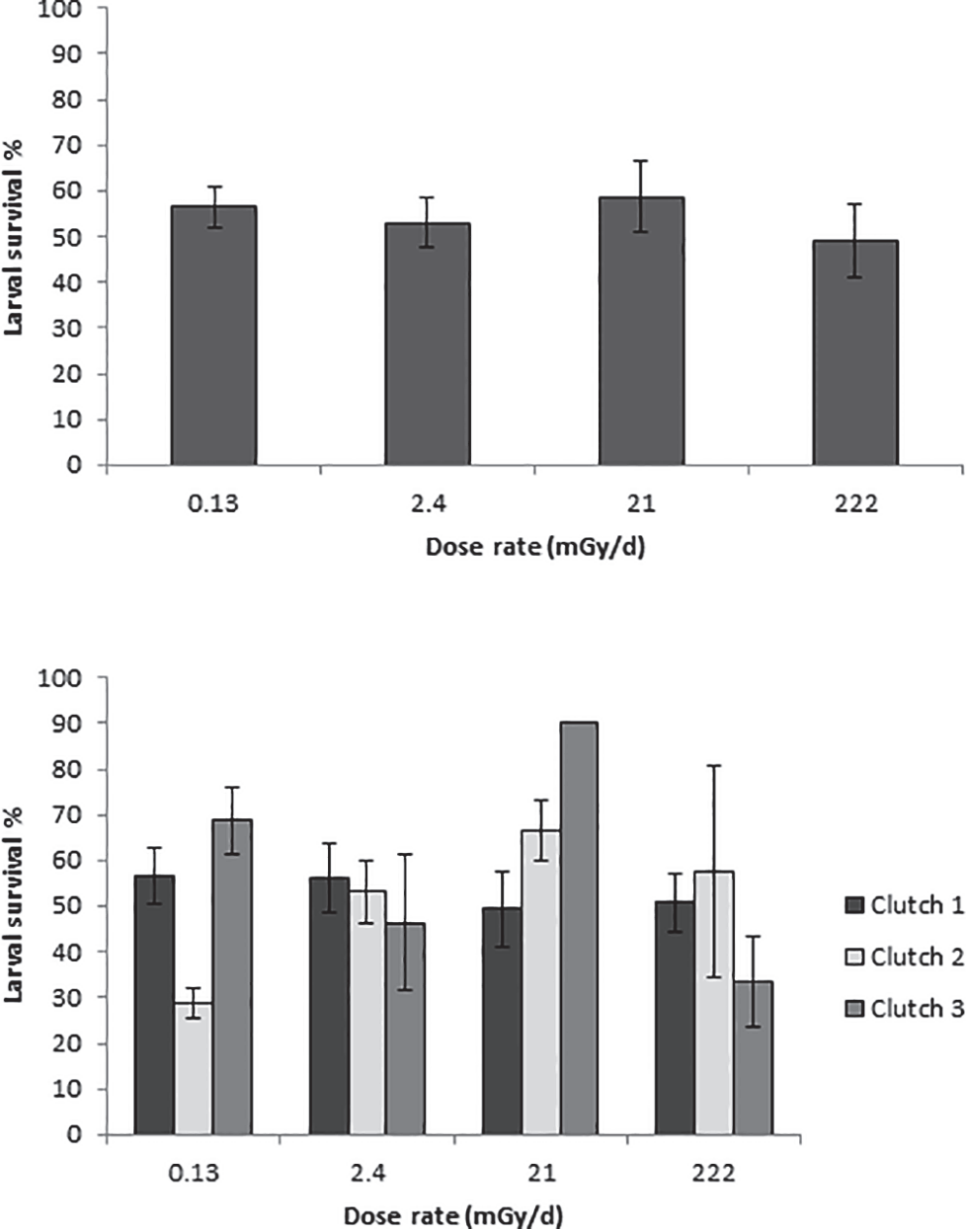
Larval survival until metamorphosis. Percent larval survival until metamorphosis of *Anaxyrus* [*Bufo*] *terrestris* tadpoles exposed to four dose rates of ^137^Cs presented as average (*top figure*) and per clutch (*bottom figure*). Because dragonfly predation caused density effects, only bucket with >20–30 tadpoles were included in the analysis. The error bars indicate standard errors of the means.

Continued low dose radiation exposure of hatchlings through larval development caused no significant difference in survival to metamorphosis among the treatments (*F*_3, 26_ = 1.39, *p* = 0.267; Fig 2) nor among the clutches (*F*_2, 26_ = 0.28, *p* = 0.756; Fig 2). Also, the interaction between treatment and clutch was not significant (*F*_6, 26_ = 1.35, *p* = 0.268). Within each clutch no significant differences were observed in survival to metamorphosis from the controls (*p* ≥ 0.198).

**Fig 3:**
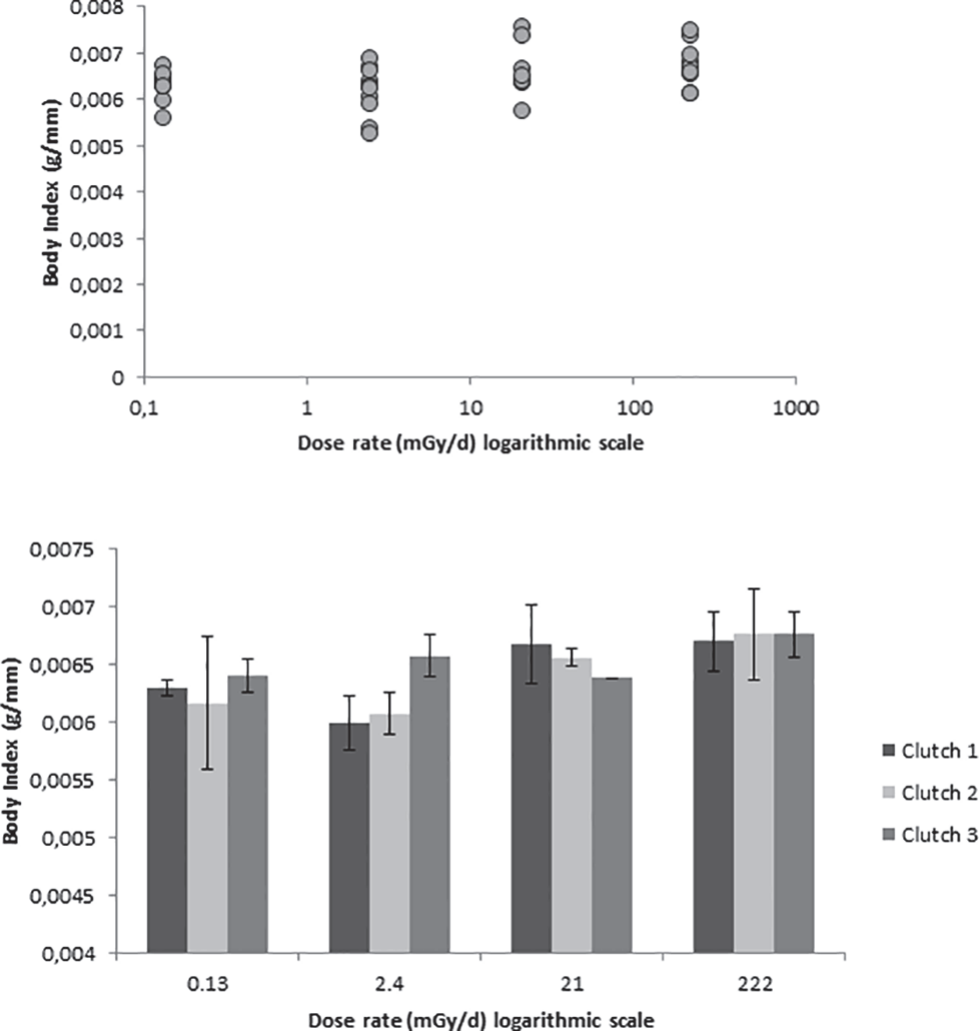
Body size after metamophosis. Body Index (mass/snout-to-vent length; g/mm) of metamorphosed *Anaxyrus* [*Bufo*] *terrestris* tadpoles exposed continuously to radiation during their larval period presented as average per bucket (*top figure*) and as average per egg clutch with standard error (*bottom figure*). The four ^137^Cs radiation treatments (0.13, 2.4, 21, and 222 mGy d^-1^) are indicated on the x-axis. Because dragonfly predation caused density effects, only buckets with >20–30 tadpoles were included in the analysis.

Body mass and body index (mass/SVL) of newly metamorphosed juveniles of *A*. *terrestris* increased linearly with radiation treatment (linear trend analysis, mass: *F*_1, 34)_ = 4.25, *p* = 0.047; index: *F*_1,34_ = 6.48, *p* = 0.016; Fig 3). SVL did not show any difference between treatments (linear trend analysis, *F*_1, 34_ = 0.82, *p* = 0.372). In addition, body index correlated with the logarithm of total dose (up to 15.8 Gy) received during the whole exposure period (*r* = 0.35, *p* = 0.032; Fig 3). When clutch was included in the analysis model, the linear effect was almost significant for index (*F*_1, 26_ = 4.15, *p* = 0.052) but not for mass (*F*_1, 26_ = 2.33, *p* = 0.139) and SVL (*F*_1, 26_ = 0.358, *p* = 0.555). There was no difference in body size (mass, SVL, and body index) between the three clutches (*F*_2, 26_ ≤ 0.25, *p* ≥ 0.779).

The length of larval development (in days) until metamorphosis was not affected by the radiation treatment (*F*_3, 26_ = 0.43, *p* = 0.733) and there was no difference in larval period between clutches (*F*_2, 26_ = 1.16, *p* = 0.333). Also, the interaction between treatment and clutch was not significant (*F*_6, 26_ = 0.69, *p* = 0.658). Within each clutch no significant differences were observed in length of larval development until metamorphosis from the controls (*p* ≥ 0.198).

**Fig 4:**
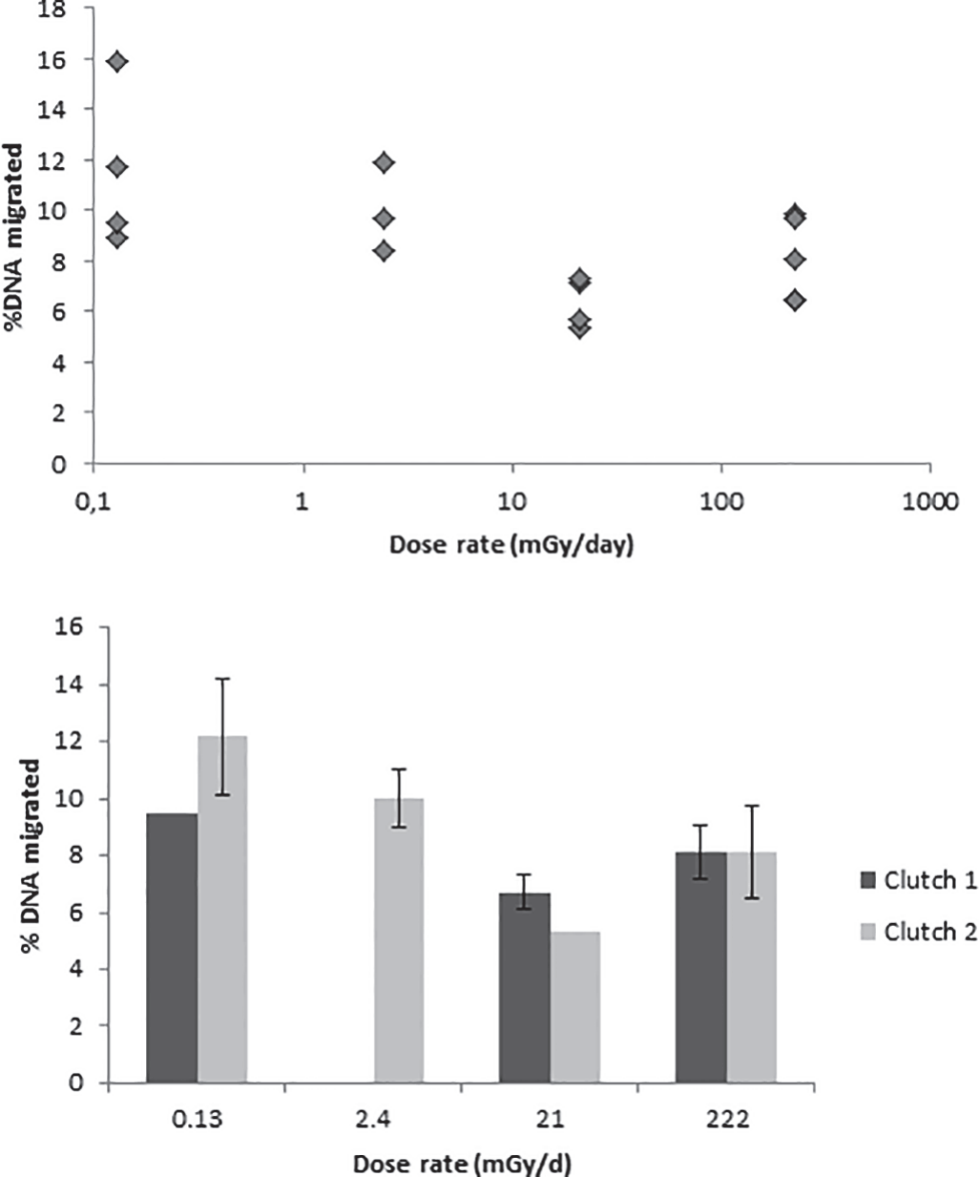
DNA damage in blood cells. Percentage of migrated DNA in red blood cells of a subsample of individuals of newly metamorphosed *Anaxyrus* [*Bufo*] *terrestris* exposed since embryos to four radiation dose rates of ^137^Cs. Results are presented as average per sample (*top figure*) and as average per egg clutch with standard error (*bottom figure*).

At the cellular level, in a subsample (N = 16) of metamorphosed *A*. *terrestris* exposed for 52 days, there was a difference in DNA damage for the radiation treatments (*F*_3, 12_ = 5.85, *p* = 0.0106; Fig 4). The percent (%) of migrated DNA in the Comet tail was lower in samples exposed to 21 mGy d^-1^ and a total dose of 1.1 Gy than in samples exposed to 0.13 mGy d^-1^ and a total dose of 0.007 Gy (Tukey’s HSD post-hoc test, *p* = 0.0095; Table 3). Thus, a lower amount of strand breaks was found in samples exposed to 21 mGy d^-1^. In addition, the decreasing DNA damage was correlated with the logarithm of increasing total dose received by the toads during the exposure (*r* = 0.58, *p* = 0.018; Fig 4).

**Table 3:** Variation in percentage of migrated DNA in blood cells.

Dose rate mGy d^-1^	Total dose (Gy) after 52 days	Variation from control samples	Standard deviation (σ)
2.4	0.125	-1.50	±1.79
21	1.1	-5.14	±1.00
222	11.5	-3.41	±1.65

Percentage of migrated DNA in red blood cells of a subsample of individuals of newly metamorphosed *Anaxyrus* [*Bufo*] *terrestris* exposed since embryos from 52 days to four radiation dose rates of ^137^Cs. Results are presented as variation from average percent migrated DNA in control samples (0.13 mGy d^-1^) with standard variation.

## Discussion

In our study, we used multiple level endpoints to assess the radiosensitivity of Southern toad (*A*. *terrestris*) during its pre-terrestrial stages of development. Body size (mass and body index (mass/SVL)) at metamorphosis increased in juvenile *A*. *terrestris* exposed since embryos to 21–32 and 222–343 mGy d^-1^ of ionizing radiation from ^137^Cs. In addition, DNA damage in red blood cells decreased in animals from clutch 1 and 2 (clutch 3 was not measured for DNA damage) after exposure to low dose rates of ^137^Cs. These results may indicate a link between a triggered growth and an increased cellular repair response of *A*. *terrestris* to low dose rate irradiation received during larval development. Body size at metamorphosis could influence later success in the reproduction of amphibians. *Ambystoma talpodieum* (mole salamander) with larger body size at metamorphosis reproduced one year earlier than individuals with smaller body size [23]. However, we do not know if the increase in body size demonstrated in the current study would be a persistent advantage and influence the success of exposed individuals in dispersal and survival out in the field or their reproduction success later in life. Hormesis responses to low contaminant concentrations have been found for many chemicals [38] and advantages or disadvantages of possible radiation hormesis has been debated for decades [39]. The results from the present study suggests that the complex effects of low dose rate ionizing radiation on amphibian populations may trigger growth and cellular repair mechanisms in exposed individuals. It is important to note the individual family variability (among the three clutches) that we observed in response of *A*. *terrestris* especially for hatching success of embryos. Previously, individual variability in response to low doses of ionizing radiation has been shown in Medaka families [40]. In that study 50% of the exposed Medaka families were more sensitive to irradiation and showed significant increases in germline mutations, while the other 50% of the families were more resistant. These findings suggest that family variability in radiosensitivity is important to include in studies of low dose effects of radiation.

Repair mechanisms are an important function in cellular physiology. A triggered, or up-regulated, repair system has been reported in studies of adaptive response; when animals are first exposed to a low dose of radiation that enhances repair of a subsequent higher dose of radiation (e.g., [41–42]. Such activated repair mechanisms might explain the lower level of DNA damage that we observed in the *A*. *terrestris* exposed to 21 mGy d^-1^ compared to the damage observed in animals exposed to 0.13 mGy d^-1^. The percentage of migrated DNA in *A*. *terrestris* exposed to 0.13 mGy d^-1^ was higher (8.9–15.9%) than measured in metamorphosed Eastern Spadefoot toads (*Scaphiopus holbrookii*) exposed to 0.13 mGy d^-1^ for 13 days in the same facility (3.5–7.2%; unpublished data from the experiment in [21]). Possibly, this difference may be due to the fact that these two species differ in radiosensitivity or that *S*. *holbrookii* were exposed for a shorter period (13 days) than *A*. *terrestris* (52 days) and that the damage caused by this low dose rate does not trigger cellular repair mechanism. Jarvis and Knowles [43] acutely exposed zebrafish to dose rates (1.2 mGy h^-1^ for 1 and 24 h) that were close to those that we used in our medium and high treatments (0.9 and 9.2 mGy h^-1^). They, however, saw an increase in DNA damage compared to controls. The different responses to low dose rate exposures may be due to dissimilarities in radiosensitivites of zebrafish and toads, or as likely, due to the acute versus chronic exposures experienced by the zebrafish and toads, respectively. Other research with amphibians documented an increase in short fragments of DNA strand breaks due to exposure from the Chernobyl accident [44]. However, it would be difficult to compare our results with the finding of that study because of the much higher radiation doses at Chernobyl, different exposures (multiple generations exposed to chronic irradiation), and different contaminants (^90^Sr and ^137^Cs). Also, it is difficult to interpret whether effects found after a radiation accident is caused by chronic exposure or whether the effects were caused by the high acute exposure situation immediately after the accident [17]. Because the alkaline Comet Assay only detects DNA damages that can be repaired, it is important to note that we did not examine if mutations or misrepair occurred in DNA of the amphibian species, which could have an impact on, for example, their reproductive success and population dynamics [45].

Hatching success of embryos is necessary for recruitment to the amphibian population. Panter [46] found a LD_50/40_ value of 0.6 Gy (acute dose) for recently fertilized eggs from *Limnodynastes tasmaniensis* (spotted grass/marsh frog), which is a lower total dose than what embryos received during four days in this study (up to 1.4 Gy). However, older embryos, stage 14–17 (as used in this study), were found to have a LD_50/40_ value of 9–10 Gy, which indicates that older embryos are more radioresistant [46]. Accordingly, results of the present study indicate that natural variation in hatching success among clutches of *A*. *terrestris* embryos (clutch 1 was significantly different from clutch 2) is much larger than the effect from low dose rate exposure from ^137^Cs. In a field study in 1993, *R*. *arvalis* (moor frog) embryos were found to have only a 10% hatching success near Lake Berdenish in the Kyshtym radioactive trace contaminated with ^90^Sr. Controls had a 45–90% hatching success (referred to in [47]). In addition, reduced hatching success of *R*. *arvalis* embryos was detected after the Chernobyl accident (referred to in [22]). Radiation damage on the male frogs’ spermatogenesis was suggested as the cause for the reduced hatching success in this case. Unfortunately, radiation doses were not reported in the two studies.

Many amphibian species lay their eggs in temporary wetlands that will dry during the summer period, which makes the length of larval period a critical parameter [26]. As little as a 15-day longer larval period enhanced the recruitment of juvenile *R*. *sphenocephala* (southern leopard frog; [25]. No significant difference was found in the length of larval period (in days) for tadpoles of *A*. *terrestris* exposed to dose rates up to 222 mGy d^-1^ during embryo development, larval period, and metamorphosis. Exposure of amphibians to higher dose rates found delayed metamorphosis at an acute exposure of 1 Gy [48]. In our experiments, tadpoles received up to 15.8 Gy during a 71-day period without signs of delayed metamorphosis.

As in the variation in hatching success of embryos, the average larval survival until metamorphosis varied greatly (30–70%; Fig 2) between clutches in controls and no effect of low dose rate exposure was detected. This variation may not be due to the low level of scatter radiation received by controls in our study because similar variation in larval survival has been measured in other studies. In experiments with copper exposed *A*. *terrestris* a larval survival of 20–60% was measured in controls [49]. In contrast, almost 80% of the control larvae of *A*. *terrestris* survived in a mesocosm study with contaminated sediment [30].

Natural stressors such as density may have a stronger effect on amphibian individuals than effects caused by low dose rate irradiation [21]. The presence of natural confounding stressors in the environment often makes it difficult to interpret radiation effects in field studies. Outdoor exposure facilities, such as the one used in this experiment, which provides a more realistic exposure scenario than laboratory exposures, may be a good alternative to further the understanding of low dose rate effects on aquatic vertebrates.

## Conclusions

Chronic exposure of *A*. *terrestris* (from embryos to metamorphosis) to low dose rate ionizing radiation resulted in increased body size at metamorphosis and decreased DNA damage in red blood cells, indicating a link between triggered growth and cellular repair response. In contrast, natural variation in hatching success of embryos between *A*. *terrestris* egg clutches was larger than the effects observed from low dose radiation exposure. Therefore, animals from different families (clutches) should be included in radiation studies to avoid under- or over-estimation of effects. Further, radiation exposures did not result in significant effects on larval survival and length of larval period. In conclusion, the complex effects from chronic radiation in the lower dose rate ranges may trigger growth and cellular repair mechanisms in amphibian larvae. Outdoor exposure facilities such as the LoDIF used in the present study may be a good approach to further the understanding of effects from environmentally relevant low dose rates because they provide more natural conditions under controlled exposure circumstances.

## Supporting Information

S1 Fig Data file. (XLSX)
